# A Case Report of Reactive Arthritis After COVID-19 AstraZeneca Vaccination

**DOI:** 10.7759/cureus.35544

**Published:** 2023-02-27

**Authors:** Nora Alalem, Naveed Yousaf

**Affiliations:** 1 Family Medicine, King Faisal Specialist Hospital and Research Centre, Riyadh, SAU

**Keywords:** side effects, sars-cov-2 vaccine, reactive arthritis, astrazeneca vaccine, covid-19 vaccine

## Abstract

In early 2021, multiple vaccinations for the coronavirus disease 2019 (COVID-19) in various immunological formulations were administered successfully to humans worldwide. Although numerous encountered side effects were expected, there were some effects that were unexpected.

We report a case of a patient who experienced a rare occurrence of reactive arthritis in the right knee joint that manifested insidiously as pain, heat, and swelling on the second day following vaccination with the Oxford-AstraZeneca COVID-19 vaccine. The patient underwent a series of investigations, confirming the suspected diagnosis and ruling out other possible diseases. The case was refractory to oral non-steroidal anti-inflammatory drugs. Thus, the treatment was shifted to intra-articular steroids. Although the treatment plan improved the symptoms of the patient noticeably, it did not resolve them.

A rare possible side effect following COVID-19 vaccination is reactive arthritis, which often occurs in young and healthy individuals with no significant comorbidities.

## Introduction

The coronavirus disease 2019 (COVID-19) has swept the world in a pandemic crisis since early March 2019. In February 2021, the first doses of the approved vaccines were made globally available. Consequently, cases of side effects from the vaccinations have been reported since, just as in Saudi Arabia. Generally, side effects of the COVID-19 vaccines range from the conventional local injection site pain, fever, malaise, and fatigue, to conjunctivitis, shortness of breath, and the more serious thrombotic events said to be associated with certain vaccine types. However, reactive arthritis (ReA) is not a commonly noted side effect. This report shows the rare side effect of COVID-19 vaccinations, which is ReA. A few case reports were found during literature search regarding ReA after COVID-19 vaccination. One case was located in the left elbow joint following Sputnik V vaccination [1]. The other case occurred after CoronaVac vaccination and affected the left knee joint [2]. Both case reports involved young and previously healthy patients with no prior injuries at the concerned sites. Another case report included two patients who were above 70 years old and took the Sinovac vaccine, with one patient experiencing arthritis in the right-hand joints and the other in the left [3]. Another two cases of reactive polyarthritis in two patients over 70 years old were reported following the CoronaVac and Sinovac vaccines [4]. Throughout the medical literature, ReA has been reported following other types of vaccines, such as influenza [5], tetanus diphtheria and pertussis [6], and hepatitis B [7].

## Case presentation

**Figure 1:**
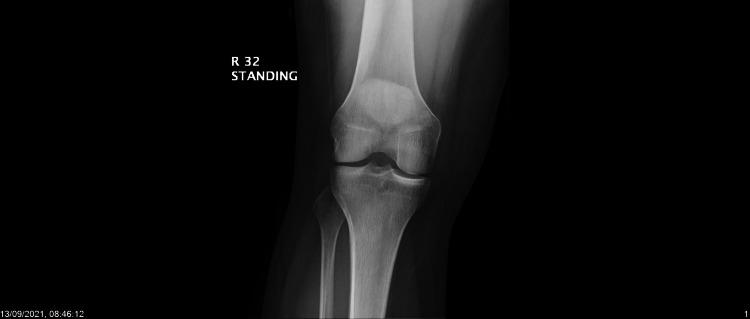
Anterior right knee plain radiograph

**Figure 2:**
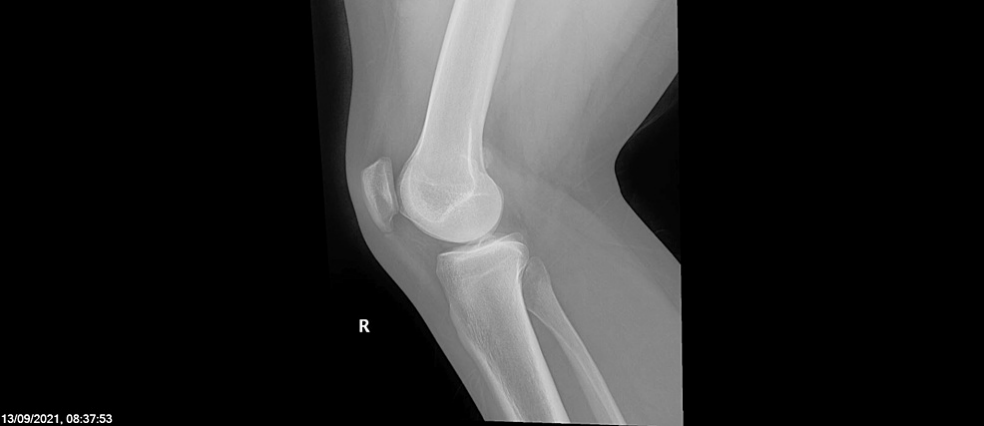
Lateral right knee plain radiograph

**Figure 3:**
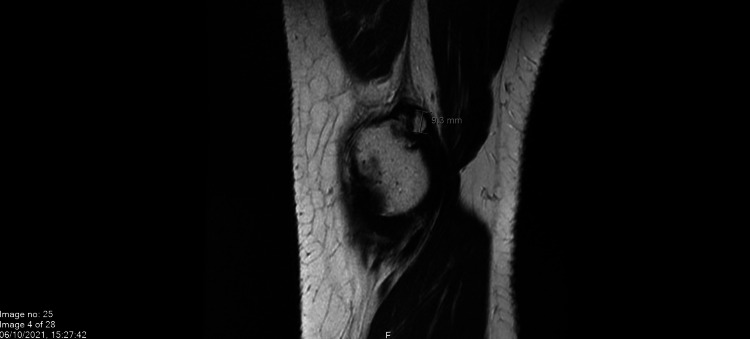
Right knee MRI

The patient is a 24-year-old healthy man who presented multiple times to the urgent care and family medicine clinic in King Faisal Specialist Hospital, Riyadh, Saudi Arabia, with a repeat complaint of pain in the right knee. One month before the last presentation of the patient, he had received the second dose of the Oxford-AstraZeneca COVID-19 vaccine. The patient reportedly experienced right knee pain three to four days after the second dose of vaccination. Over the course of the following month, the patient visited the clinic a couple of times, solely due to the right knee pain. Documented physical examination did not show any limitation in the range of motion, effusion, or erythema at the time of examination. A radiograph of the knee showed a possible old avulsion injury to the dorsal aspect of medial epicondyle (Figures 1, 2). An MRI showed an old avulsion injury of adductor magnus tendon with mild joint effusion, as well as imaging features of patellar mall tracking syndrome (Figure 3).

In September 2021, the patient presented to the urgent care clinic with a complaint of pain in the right knee that exhibited swelling and was warm to the touch. The patient denied fever, dysuria, urethral discharge, and eye pain. On examination, there was clear asymmetry between both knees; the medial aspect of the right knee was more pronounced. There was mild erythema and heat. Range of motion remained intact, but movement was mildly painful. Initial laboratory test results showed leukocytosis of 12.22 10^9^/L (high) with left shift: neutrophil count of 70.8 10^9^/L (high), absolute neutrophil count of 8.6 10^9^/L (high), and lymphocyte count of 20.6 109/L (low). The C-reactive protein level was 5.7 mg/L (high). Tests for HLA-b27, rheumatoid factor, anti-citrullinated C peptide, anti-nuclear antibodies, and anti-streptolysin O titer were all negative. Blood culture showed no growth. The SARS-CoV-2 post-vaccine anti-spike 1 IgG was 1,810 u/mL.

On urine analysis, rapid chlamydia, gonorrhea, and urine culture were all negative. A repeat radiograph of the right knee showed persistent possible old avulsion injury to the dorsal aspect of medial epicondyle along with knee effusion. A radiograph of the sacroiliacs did not show any abnormality.

Joint aspiration was performed to rule out septic arthritis, and the results showed yellow turbid fluid, with a white blood cell count of 2,425 10^9^/L (high), neutrophils of 45% (high), lymphocyte of 26% (normal), protein of 45 g/L, glucose of 5.7 mmol/L, and red blood cell count of 6,225 10^9^/L (high). Gram staining revealed no organisms as did the initial acid-fast stain. No crystal formation was identified.

The patient’s pain was refractory to oral non-steroidal anti-inflammatory drugs (NSAIDs) ibuprofen 600 mg and naproxen 500 mg. Due to possible side effects and non-responsiveness of oral NSAIDs, the treatment was shifted to an intraarticular steroid. A 50 mg injection of triamcinolone was given intra-articularly. After three days, the patient was followed up over the phone, and resolution of swelling and improvement in pain was reported by the patient. However, the symptoms did not completely resolve upon one-week follow-up after the injection. A rheumatologist was involved at this point, and magnetic resonance imaging was performed, which confirmed the old avulsion injury of adductor magnus with mild joint effusion.

Upon follow-up of the patient 10 months after the initial encounter, the pain and swelling returned gradually. No cutaneous or gastrointestinal symptoms developed during that time. Orthopedic surgery was consulted for the management of chondromalacia patella and intra-articular loose body as the possible causes. The patient started physical therapy with not much improvement.

## Discussion

The findings are consistent with ReA as the possible diagnosis. The onset of symptoms was around three days after the second dose of Oxford-AstraZeneca COVID-19 vaccine. In the other reported case, ReA occurred seven days after Sputnik V vaccination in the left elbow joint.

ReA seems to occur in large joints. In the other case, ReA occurred in the left knee joint on day 3 post-vaccination with CoronaVac vaccine [2]. This suggests that no specific type of COVID-19 vaccine is responsible for the side effect, rather an immunological reaction to any of the COVID-19 vaccines is the likely cause. In the case where CoronaVac vaccine was administered, the patient required an intra-articular injection of 1 mL of betamethasone, which improved the symptoms of swelling and erythema within two days and resolved completely at one month after. However, the pain persisted. Similarly, in our case, swelling resolved within three days of administration of intra-articular steroid injection and pain had improved but still persisted.

In our patient, there is a possibility that an old injury in the right knee acquired during childhood had damaged the joint integrity and might have predisposed the patient to ReA. However, in the other reported cases, no prior injuries were identified during history taking or imaging.

The natural history and presentation of this case highly suggest ReA and the most recent preceding event was the COVID-19 vaccination. A clear causative relationship cannot be established; however, it is highly suggested through these reported cases (Table 1).

**Table 1:** Comparison between cases found in the literature and the presented case report CRP, C-reactive protein; ESR, erythrocyte sedimentation rate; MCP, metacarpophalangeal; NSAIDS, non-steroidal anti-inflammatory drugs; PIP, proximal interphalangeal; PT, physical therapy

Case/Reference	Vaccine	Joint	Onset	Intervention	Follow-up	Age (years)/gender	CRP(mg/L)	ESR(mm/hr)
Case 1 [1]	Sputnik-V	Left elbow joint	Day 5 post-vaccine dose #2	NSAIDs, PT, Injection	1 month, arthralgia on active motion present	58/male	14	18
Case 2 [2]	CoronaVac	Left knee joint	Day 3 post-vaccine dose #1	NSAIDs, PT, injection	1 month	23/female	15	32
Case 3a [3]	Sinovac	Right wrist, 2nd and 3rd MCP and PIP joints	Day 2 post-vaccine dose #1	Tapering oral steroids regimen and injection	1 week, resolved	74/female	20	84
Case 3b [3]	Sinovac	Left wrist, MCP and PIP joints	Day 7 post-vaccine dose #2	Tapering oral steroids regimen and injection	Unclear, resolved	76/male	11.2	85
Case 4 (this case report)	AstraZeneca	Right knee joint	Day 3 post-vaccine dose #2	NSAIDs, PT, injection	1 week with initial improvement, 10 months, returning mono-arthritis	24/male	5.7	Nil

## Conclusions

ReA after COVID-19 vaccine administration should be considered in any case presenting with mono-arthritis following vaccination. It is important that septic arthritis must be ruled out first. Cases of ReA included in this report might have required more than just oral NSAIDs for resolution. Sequalae of the disease may take time to resolve and may not completely resolve. Continuous physical therapy and rheumatological involvement are suggested in resistant cases.
